# People’s Intuitions About Innateness

**DOI:** 10.1162/opmi_a_00029

**Published:** 2019-10-01

**Authors:** Iris Berent, Melanie Platt, Gwendolyn M. Sandoboe

**Affiliations:** Department of Psychology, Northeastern University; Department of Psychology, Northeastern University; Department of Psychology, Northeastern University

**Keywords:** innateness, core knowledge, dualism, essentialism

## Abstract

Few questions in science are as controversial as the origins of knowledge. Whether knowledge (e.g., “objects are cohesive”) is partly innate has been debated for centuries. Here, we ask whether our difficulties with innate knowledge could be grounded in human cognition itself. In eight experiments, we compared reasoning about the innateness of traits that capture knowledge (cognitive traits) with noncognitive (sensorimotor and emotive) traits. Experiments 1–4 examined adult and infant traits; Experiment 5 presented detailed descriptions of published infant experiments. Results showed that people viewed cognitive traits as less likely to be innate in humans—the stronger the association with “thinking,” the lower the rating for “innateness.” Experiments 6–8 explored human, bird, and alien traits that were presented as innate. Participants, however, still considered cognitive traits as less likely to emerge spontaneously (i.e., be innate). These results show that people are selectively biased in reasoning about the origins of knowledge.

## INTRODUCTION

The origin of knowledge is one of the most controversial questions in the history of ideas. At stake is whether knowledge (e.g., the notion of an “object”) is induced from experience, or whether certain cognitive concepts and principles are innate in humans. These debates have been raging since the times of the ancient Greeks, and they show no sign of abating in the current scientific literature.

Why is innateness such a hard question? There is no doubt that the innateness challenge is partly due to the complexity of the problem at hand—the intricacy of human cognition and its mostly unknown instantiation in the brain. But perhaps the reasons for the stalemate lie not only with the topic of inquiry but also with the human inquirer. Here we ask whether our understanding of innateness could be constrained by systematic biases that shape how people reason about innate knowledge.

The possibility that people are not impartial to innateness has not gone unnoticed. Cosmides and Tooby (1994) have argued that people suffer from “instinct blindness,” and Pinker (2002) has warned against the denial of human nature. But these assertions are based on anecdotal evidence. The question thus remains whether people are indeed blind to innateness—no previous study has examined this possibility. Moreover, no research has asked whether our blindness to innateness is particularly acute when it comes to knowledge (with the exception of Wang & Feigenson, 2019, published in this issue).

Knowledge, here, refers to tacit concepts and principles. Examples of putative innate knowledge include tacit principles of core physics (e.g., “objects are cohesive”; Spelke & Kinzler, 2007) and core psychology (e.g., “agents’ actions are determined by their mental states”; Baron-Cohen, Leslie, & Frith, 1985), as well as their constituent concepts (*object*; *agent*). Our research asks whether people are reluctant to accept that knowledge can be innate.

There are various reasons to believe that the notion of innate knowledge might present a special challenge to intuitive human cognition, reasons we explore in the General Discussion. Our present inquiry, however, does not seek to explain *why* people are biased against innate knowledge, and we certainly do not intend to determine whether knowledge *is* in fact innate. Instead, we strictly ask whether people are guilty as charged—are we systematically biased in evaluating the origins of our knowledge?

To this end, we examine reasoning about the origins of various types of cognitive traits that capture knowledge and compare them to sensory, emotional, and motor traits. We refer to these traits generically as *cognitive*, but our interest is strictly in innate knowledge (e.g., “objects are cohesive”), as opposed to “knowhow” (e.g., playing the violin) or “horizontal” faculties (e.g., attention, memory, and problem solving).

In this investigation, we define “innate” psychological traits as psychological primitives—ones that are acquired in the normal course of development without relying on other psychological processes, most notably, learning (Samuels, 2004, 2007). To gauge laypeople’s reasoning about innateness, we thus asked participants to reflect on the tendency of psychological traits to emerge spontaneously in all members of the species and their early onset in development (a hallmark of many innate traits). We also asked people to explicitly indicate whether or not cognitive traits are innate.

We conducted two sets of experiments. In one set, we presented people with various traits—both cognitive and noncognitive—and asked them to reason about their innateness. To determine whether reasoning about innateness is *biased*, we next presented people with detailed vignettes, stating that all traits in question *are* innate. Of interest is whether people are reluctant to view cognitive traits as innate despite evidence that is clearly indicative of innateness.

## REASONING ABOUT HUMAN COGNITIVE TRAITS

### Experiments 1–2: Rating Human Traits

Experiments 1–2 each presented people with a list of human traits. Experiment 1 featured adult traits that have been documented cross-culturally; Experiment 2 featured infant traits. In each case, half of the traits were cognitive and half were noncognitive, either motor and emotive, or motor and sensory (in Experiments 1–2, respectively). For example, adult cognitive traits included “forming sentences” and “having a concept of ‘person’”; infant cognitive traits included “Expecting objects to move as connected wholes (e.g., without disintegrating).”

To ensure that these traits are perceived as indicative of cognition, we asked one group of participants to classify the traits into three bins (e.g., “thinking,” “emotion,” or “action”). A second group of participants evaluated the innateness of these traits, operationalized as their propensity to emerge spontaneously, in a “desert island” situation.

We examined three questions. First, do people view these cognitive traits as ones that are indicative of “thinking”? Second, do people believe that cognitive traits are less likely to emerge spontaneously (i.e., be innate)? And third, is the rating of a trait for innateness associated with its classification as “thinking”?

### Experiment 1: Rating Adult Human Traits

#### Methods

##### Participants

Two groups of participants (*N* = 20 each) were each assigned to the trait classification and innate rating tasks. In this and all subsequent experiments, participants were adult English speakers, recruited through Amazon Mechanical Turk.

##### Materials and Procedure

The materials consisted of a randomized list of 80 human traits—half were cognitive—and half were noncognitive traits, either emotive (20 traits) or motor (20 traits).

The trait list was presented to two groups of participants. One group classified each trait into one of three bins—“thinking,” “action,” or “emotion.” A second group rated the traits for innateness. Specifically, people indicated on a 1–7 scale (1 = very unlikely; 7 = very likely) how likely it is that a person would exhibit this trait spontaneously, in a desert island situation, even if they did not have the opportunity to learn it from others. For additional method details, see the Supplemental Materials; Berent, Platt, & Sandoboe, 2019b, which include the stimulus materials and instructions for this and all subsequent experiments in Appendices 1–2.

#### Results

##### Trait Classification

To determine whether most classification responses were congruent with the intended category (e.g., “thinking,” for cognitive traits), we first computed the congruence scores for cognitive and noncognitive traits (noncognitive traits were averaged over the “motor” and “emotive” subcategories). We then compared the proportion of “congruent” responses against chance (.5) using a mixed-effects logistic regression model (with participants and items as random effects). Results demonstrated that ratings of cognitive (*M* = 0.79, β_0_ = 2.14, *Z* = 5.00, *SE* = 0.43, *p* < .0001) and noncognitive (*M* = 0.90, β_0_ = 3.58, *Z* = 7.58, *SE* = 0.47, *p* < .0001) traits were congruent with the intended category.

##### Innateness Rating

**Figure 1.**
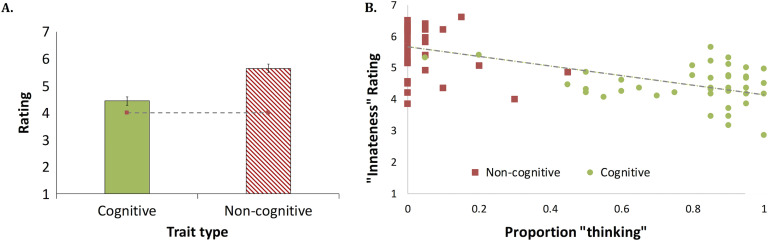
**The presumed innateness of adult human traits (A) and its association with “thinking” response (B).** In this and all subsequent experiments, the scale’s midpoint is indicated by a dashed line. Error bars are 95% confidence intervals for the difference between the means. Scatter symbols indicate individual traits.

We next examined the rating of these traits for innateness (see Figure 1A). People rated noncognitive traits significantly higher than the scale’s “neutral” midpoint, 4; *M* = 5.65, *t*_1_(19) = 7.91, *p* < .0001, *d* = 1.77; *t*_2_(39) = 13.37, *p* < .0001, *d* = 2.11. For cognitive traits, by contrast, this difference was not significant across participants, *M* = 4.44, *t*_1_(19) = 1.68, *p* < .11, *d* = 0.38; *t*_2_(39) = 4.58, *p* < .0001, *d* = 0.72. A matched sample *t* test further showed that cognitive traits were rated as less likely to emerge spontaneously compared to noncognitive traits, and these results were significant across participants, *t*_1_(19) = 7.61, *p* < .0001, *d* = 1.10, and items, *t*_2_(78) = 7.76, *p* < .0001, *d* = 1.73.

##### The “thinking”–“innateness” link.

Finally, we examined whether reasoning about the innateness of cognitive traits is linked to their association with “thinking” (see Figure 1B). To this end, we correlated the rated propensity of each of our 80 traits to emerge spontaneously (our gauge of “innateness”) with its classification as “thinking.” The correlation was highly significant and negative, *r*(78) = −.680, *p* < .0001—the stronger the association of a trait with “thinking,” the less likely it was to be seen as spontaneously emerging (i.e., innate).

### Experiment 2: Rating Infant Human Traits

#### Method

##### Participants

Two groups of participants (*N* = 20 each) were assigned to the trait-classification and innateness-rating tasks.

##### Materials and Procedure

The materials consisted of a randomized list of 32 infant traits: 16 cognitive and 16 noncognitive (10 motor and 6 sensory traits). Participants in the classification task assigned each trait into one of three bins (“thinking,” “action,” or “sensation”). In the innateness-rating task, participants indicated “how likely it is that an infant would exhibit this behavior spontaneously, without being shown or taught by a parent, caregiver, or any other person” (1 = very unlikely; 7 = very likely).

#### Results

##### Trait Classification

We first confirmed (via mixed-effects logistic regression) that the majority of classification responses were congruent with the intended category (e.g., “thinking,” for cognitive traits), and this was the case for both cognitive, *M* = 0.71, β_0_ = 1.33, *Z* = 2.78, *SE* = 0.48, *p* = .005, and noncognitive, *M* = 0.65, β_0_ = 0.88, *Z* = 2.15, *SE* = 0.41, *p* = .03, traits.

##### Innateness Rating

**Figure 2.**
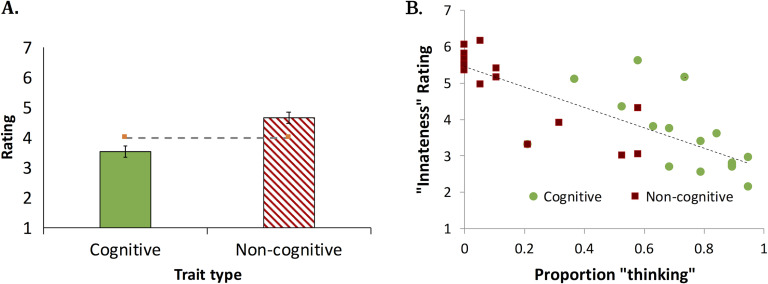
**The presumed innateness of infant traits (A) and its association with “thinking” response (B)**. Scatter symbols indicate individual traits.

Figure 2A plots participants’ ratings for the propensity of cognitive and noncognitive traits (motor and sensory) to emerge in infants. Noncognitive traits were rated significantly higher than the scale’s “neutral” midpoint (4), *M* = 4.67, *t*_1_(19) = 4.44, *p* < .0003, *d* = 0.99; *t*_2_(15) = 3.38, *p* = .004, *d* = 0.85, but that was not the case for cognitive traits, *M* = 3.54, *t*_1_(19) = −2.04, *p* = .055, *d* = −0.46; *t*_2_(15) = −1.78, *p* = .095, *d* = −0.45. Critically, cognitive traits were rated as significantly less likely to emerge spontaneously compared to noncognitive traits, *t*_1_(19) = 5.94, *p* < .0001, *d* = 1.27; *t*_2_(30) = −3.66, *p* < .001, *d* = 0.81.

##### The “thinking”–“innateness” link.

We next examined whether the perceived propensity of a trait to emerge spontaneously (a gauge of innateness) is associated with its classification as “thinking” (see Figure 2B). The correlation was negative and highly significant, *r*(30) = −.793, *p* < .0001—the stronger the association of the trait with “thinking,” the less likely it was to be viewed as innate.

### Experiments 3–4: The Innateness of Adult and Infant Traits (Forced Choice)

Experiments 1–2 demonstrate that people believe that cognitive traits are less likely to emerge spontaneously relative to noncognitive traits. To further clarify people’s innateness intuitions, in Experiments 3–4, we next asked people to explicitly indicate whether or not these traits are inborn. Experiment 3 featured the adult traits; Experiment 4 presented the infant traits.

#### Method

Experiments 3–4 presented two groups of participants (*N* = 20 each) with the adult and infant traits from Experiments 1–2, respectively. Participants were asked to make a forced choice as to whether each trait is inborn.

#### Experiment 3 (Adult Traits): Results and Discussion

We first compared the mean response for each trait type against chance (.5) using a mixed-effects logistic regression model; another model contrasted responses to cognitive and noncognitive traits (with random intercepts by subject and items, and random slopes for trait type by subjects and items).

**Figure 3.**
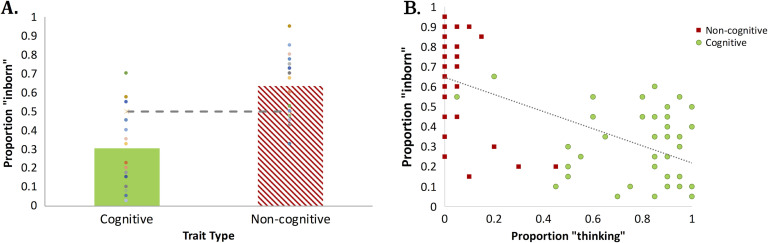
**The presumed innateness of adult traits (A) and its association with “thinking” response (B).** In Panel A, scatter symbols indicate the responses of individual participants; in Panel B, they reflect individual traits.

Noncognitive traits were rated significantly higher than chance (*M* = 0.63, β_0_ = 0.84, *Z* = 2.55, *SE* = 0.33, *p* = .01, see Figure 3). Cognitive traits, by contrast, were rated reliably lower than chance (*M* = 0.30, β_0_ = −1.25, *Z* = −3.61, *SE* = 0.35, *p* < .0004). This result demonstrates that people believe that cognitive traits are not innate.

A comparison of cognitive and noncognitive traits further confirmed that people were less likely to view cognitive traits as innate than noncognitive traits (β_1_ = 2.09, *Z* = 5.75, *SE* = 0.36, *p* < .0001). The correlation between the rating of the traits for “innateness” and “thinking” (from Experiment 1) was highly significant, *r*(78) = −.638, *p* < .0001.

#### Experiment 4 (Infant Traits): Results and Discussion

**Figure 4.**
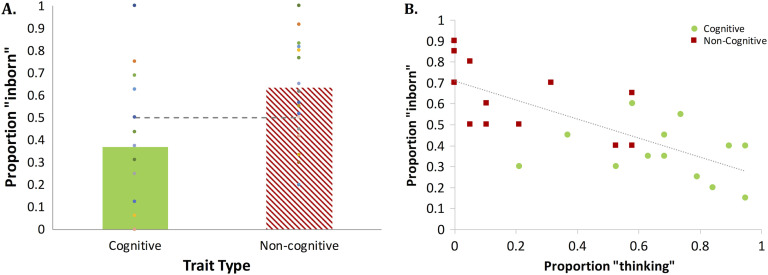
**The presumed innateness of infant traits (A) and its association with “thinking” response (B).** In Panel A, scatter symbols indicate the responses of individual participants; in Panel B, they reflect individual traits.

Responses in Experiment 4 were analyzed as described in Experiment 3. A comparison of the “inborn” responses against chance showed that people believed that cognitive traits are not inborn in infants (*M* = 0.36, β_0_ = −0.85, *Z* = −2.03, *SE* = 0.42, *p* = .04), whereas noncognitive traits were considered innate (*M* = 0.66, β_0_ = 0.94, *Z* = 2.55, *SE* = 0.37, *p* = .01, see Figure 4). Additionally, cognitive traits were rated as less likely to be inborn relative to noncognitive traits (β_1_ = 1.78, *Z* = 4.11, *SE* = 0.43, *p* < .0001). The rating of the traits for “innateness” correlated significantly with their rating for “thinking” (from Experiment 2), *r*(30) = −.761, *p* < .0001.

### Experiment 5: Infant Experiments

Experiments 3–4 showed that people believe that cognitive traits are unlikely to be innate. It is possible, however, that people are not inherently biased against innate knowledge. Rather, people do not understand how one could evaluate the cognitive capacities of infants, as they are unaware of the experimental methods of infant research. To address this possibility, in Experiment 5, we presented people with detailed vignettes, adapted from four published infant experiments (complete with methods, rationale, and predictions), and elicited a binary yes/no response as to whether those traits are present in newborns.

Two of the vignettes clearly captured knowledge. One vignette concerned newborns’ rudimentary numeric cognition—their capacity to match the number of sounds and dots (Izard, Sann, Spelke, & Streri, 2009). A second vignette described infants’ moral preferences—the preference of 3-month-olds for “helpers” over “hinderers” (Hamlin, Wynn, & Bloom, 2010). The third scenario concerned newborns’ preference for syllable structure (e.g., blog > lbog; Gómez et al., 2014); since this preference could be based on either abstract linguistic knowledge or sensorimotor restrictions, this case was intermediate between cognition and sensation. The fourth vignette, by contrast, featured an unambiguous emotional trait—the preference for happy vs. angry faces (shown in 7-month-old infants, Datyner, Henry, & Richmond, 2017). If people are reluctant to view cognitive traits as innate, then they should conclude that infants are unlikely to exhibit knowledge of number and moral preferences. Emotions, on the other hand, will be viewed as innate (Lindquist, Gendron, Oosterwijk, & Barrett, 2013).

#### Methods

Twenty participants took part in the experiment. The materials described four published experiments, examining infants’ sensitivity to four traits: language, number, moral preferences, and emotion. Participants made a binary yes/no response as to whether newborns will be likely to exhibit sensitivity to the relevant distinction and provided a brief explanation for their response.

#### Results

**Figure 5.**
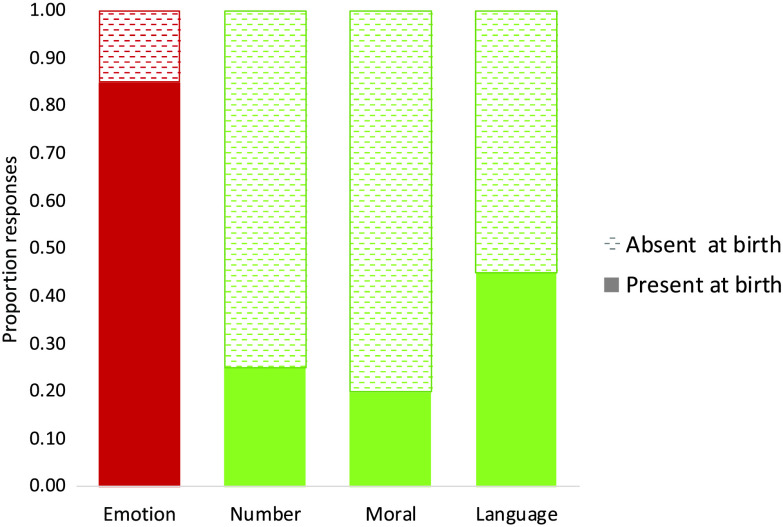
The presumed spontaneous emergence of cognitive and emotive traits of newborn infants.

As predicted, people believed that the two unambiguous cognitive traits—numeric cognition (*M* = 0.25, *p* = .04) and moral cognition (*M* = 0.20, *p* = .01)—are absent at birth (see Figure 5), and binomial tests indicated that the frequency of these responses differed significantly from chance. For the language task, responses were at chance (*M* = 0.45, *p* = .82). Most participants who asserted that newborns prefer syllables like “blog” mentioned the sound of these syllables in their explanations; this is in line with the possibility that people attribute syllable structure to sensory, rather than cognitive constraints. In contrast, in the emotion task, people asserted that newborns will show a preference for smiling faces (*M* = 0.85, *p* = .004), and their justifications often appealed to emotion (e.g., “I think infants can distinguish between happy and sad faces”; for all justifications, see the Supplemental Materials; Berent et al., 2019b, Appendix 3).

## REASONING ABOUT INNATE COGNITIVE TRAITS

Why do people assert that cognitive traits are less likely to be innate? One possibility is that this belief is due to innocent misinformation. People, indeed, know that young infants can smile and move, but few are aware of research on infant cognition. Accordingly, participants have no reason to suspect that some cognitive traits are present at birth. On an alternative account, participants’ behavior reflects an active *bias*. People, in this view, are not simply unaware of innate knowledge. Rather, they are unwilling to entertain the possibility that some knowledge is innate.

To adjudicate between these possibilities, in Experiments 6–8 we presented people with detailed vignettes featuring a single trait. Cognitive traits underscored information structure (e.g., the rule-based structure of communication), whereas motor traits featured physical action. To reduce interference from relevant experience, Experiments 6–7 featured bird and alien species; Experiment 8 reexamined the basis of the human capacity for language. Critically, participants were provided with evidence indicating that *all traits are innate*. Of interest is whether participants would still consider cognitive traits as less likely to emerge spontaneously.

### Experiment 6: Bird Traits

#### Methods

##### Participants

Forty participants took part in this experiment.

##### Materials and Procedure

The vignettes featured four traits of distinct bird species—two behaviors were related to flying, the other two concerned singing. The vignettes were arranged in matched pairs (see the Supplemental Materials; Berent et al., 2019b, Appendix 1); one pair member featured a cognitive trait (e.g., structure of the swamp sparrow’s song); its matched member featured a motor trait (e.g., the quail’s head-bobbing, produced during singing). Participants were explicitly informed that researchers believe that each of the four traits are likely inborn, universal to all members of the species, and early emerging in development. Participants were next invited to imagine that the bird’s fertilized eggs were incubated in isolation; the hatched chicks would be well cared for, but without exposure to the behavior at hand. They were asked to rate on a 1–7 scale (1 = very unlikely; 7 = very likely) how likely the bird is to produce the relevant behavior once it matures.

#### Results and Discussion

**Figure 6.**
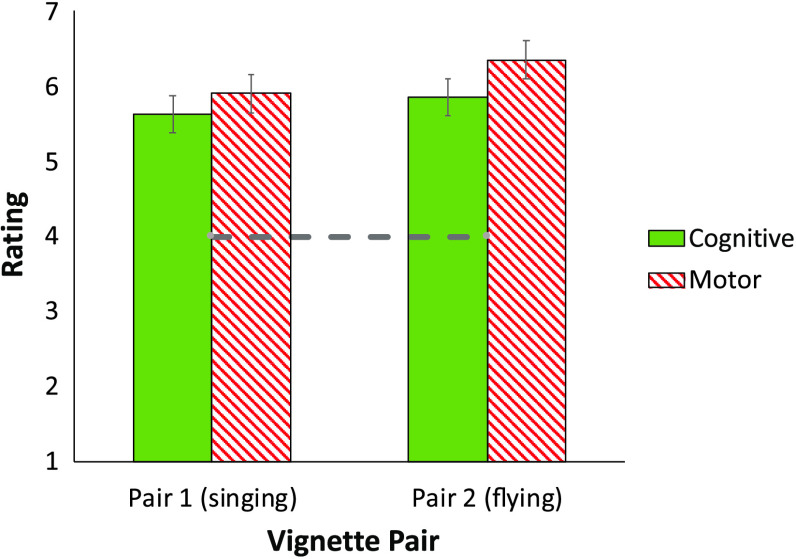
The presumed innateness of cognitive and motor traits of birds (in Experiment 6).

Figure 6 plots participants’ rating of the propensity of bird traits to emerge spontaneously (a gauge of their perceived innateness). We first confirmed that cognitive, *M* = 5.74, *t*(39) = 8.86, *p* < .0001, *d* = 1.40, and noncognitive, *M* = 6.13, *t*(39) = 14.64, *p* < .0001, *d* = 2.31, traits were each rated higher than the scale’s “neutral” midpoint. This suggests that people heeded the instructions, indicating that both traits are innate. Of interest, though, is whether they were equally likely to accept these facts in both conditions.

A 2 Trait (Cognitive/Motor) × 2 Vignette pair ANOVA yielded a reliable effect of Trait, *F*(1,39) = 7.05, *p* = .01; $\eta_{\text{partial}}^{2}$ = .153. There was also a marginally significant effect of Vignette pair, *F*(1, 39) = 4.07, *p* = .05; $\eta_{\text{partial}}^{2}$ = .094, as people gave higher innateness ratings to the vignette pair concerning flying compared to those concerning singing. The Trait × Vignette interaction, however, was not significant (*F* < 1). Thus, despite being explicitly informed that all bird traits are early emerging, adaptive, and likely inborn, people viewed cognitive traits as less likely to emerge spontaneously (relative to noncognitive traits).

### Experiment 7: Alien Traits

#### Method

##### Participants

Twenty participants took part in this experiment.

##### Materials and Procedure

The materials consisted of four vignettes arranged in pairs, matched for narrative structure and length. One pair contrasted the cognitive capacity of the alien species to communicate using light signals and their motor capacity for locomotion using a circular movement style. The second pair of vignettes contrasted the cognitive ability of the aliens to reckon their navigational path by integrating multiple cues, and their motor capacity for flying by “bounding.” Each such trait was explicitly described as innate, universal, and early emerging in development.

Participants were asked to imagine that infant members of the species were reared in isolation (fully cared for) and rate (on a 1–7 scale) how likely each trait is to emerge in those aliens as they grew into adults.

#### Results

**Figure 7.**
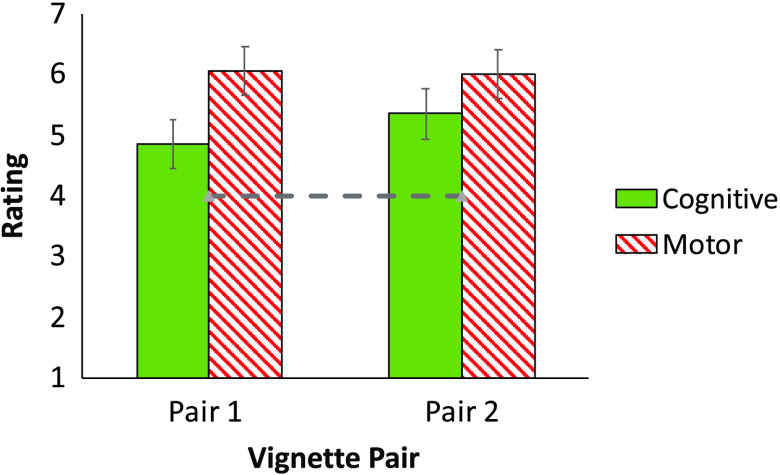
The presumed innateness of cognitive and motor traits in aliens (in Experiment 7).

A comparison of the means against the scale’s “neutral” midpoint confirmed that people thought cognitive, *M* = 5.10, *t*(19) = 2.81, *p* = .01, *d* = 0.63, and noncognitive, *M* = 6.03, *t*(19) = 8.89, *p* < .0001, *d* = 1.99, traits would each emerge spontaneously in aliens (see Figure 7). This is only expected given that the instructions explicitly presented both traits as innate. But an inspection of the means suggests that the innateness ratings were nonetheless lower for cognitive traits.

A 2 Trait (Cognitive/Motor) × 2 Vignette pair ANOVA yielded a reliable effect of Trait, *F*(1, 19) = 5.59, *p* = .03; $\eta_{\text{partial}}^{2}$ = .227. No other effects were significant (all *F* < 1). Thus, people viewed cognitive traits of aliens as less likely to emerge spontaneously.

### Experiment 8: Human Language

#### Methods

##### Participants

Forty participants took part in this experiment.

##### Materials and Procedure

The materials consisted of two matched vignettes, describing a restriction on the sequencing of linguistic elements. One story described an abstract cognitive constraint on the syntactic sequencing of actors and actions (e.g., “dogs bark” vs. “bark dogs”); the other story described a motor articulatory constraint on the sequencing of speech sounds (e.g., blog vs. lbog). Each such restriction was explicitly described as universal and spontaneously emerging (without learning). Each story was assigned to a distinct subgroup of participants (*N* = 20 each). Participants rated (on a 1–7 scale) how likely it is that the restriction will emerge spontaneously among a group of children who are raised together, fully cared for, but devoid of exposure to spoken language.

#### Results

**Figure 8.**
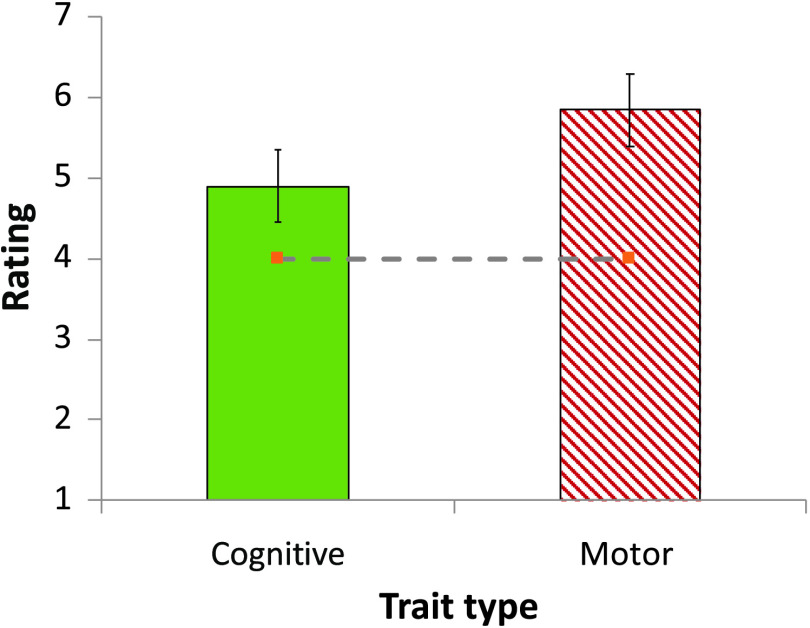
The presumed innateness of cognitive and motor aspects of human language (in Experiment 8).

Although both traits were presented as innate, participants viewed the cognitive syntactic restriction as significantly less likely to emerge spontaneously than the articulatory motor restriction on sound combinations, *t*(38) = 2.12, *p* = .04, *d* = 0.67 (see Figure 8). It is unlikely that people simply ignored the instructions. Indeed, cognitive, *M* = 4.9, *t*(19) = 2.71, *p* = .01, *d* = 0.61, and noncognitive, *M* = 5.85, *t*(19) = 6.14, *p* < .0001, *d* = 1.37, traits were each rated higher than the scale’s midpoint. Nonetheless, people were reliably less likely to accept the conclusion that syntactic cognitive traits will emerge in humans spontaneously.

## GENERAL DISCUSSION

This investigation evaluated how laypeople reason about the innateness of cognitive traits. Across eight experiments, we found that people consistently consider traits associated with “thinking” as less likely to be inborn.

Experiments 1–2 found that people rated cognitive traits as less likely to emerge spontaneously in both adults and infants. Experiments 3–4 explicitly asked participants whether psychological traits are inborn. To further encourage participants to contrast innate and acquired traits, we elicited a forced choice. Here, people responded that noncognitive traits of adults and infants are inborn, but cognitive traits are not. Experiment 5 observed that people were disinclined to believe that infants exhibit certain cognitive traits when they were presented with detailed descriptions of infant research. Finally, Experiments 6–8 presented people with detailed descriptions of various traits of birds, aliens, and humans, suggesting each such trait is innate. But despite clear evidence for innateness, people remained less likely to entertain the possibility that cognitive traits will emerge spontaneously (i.e., be innate) relative to noncognitive traits.

The resistance to innateness was specific to cognitive traits that encompass knowledge. Participants in Experiments 1–4 consistently rated noncognitive traits higher than the scale’s neutral midpoint, but for cognitive traits, they were either neutral (in Experiments 1–2) or actively averse to innateness (in Experiments 3–4). Moreover, the reluctance to view a given trait as innate was negatively correlated with its association with “thinking.” Likewise, participants in Experiment 5 clearly viewed emotions as innate, but they selectively rejected the innateness of numeric and moral cognition. Experiments 6–8 documented the same phenomenon in detailed, closely matched vignettes featuring cognitive and motor traits. Whether cognitive traits are in fact innate is a critical question that falls beyond the scope of our research. Our results, however, do suggest that people are reluctant to entertain this possibility.

Why are people resistant to the notion of innate knowledge? Our results are not readily explained by previous accounts of antinativism. One such proposal asserts that people are generally resistant to nativism because they are concerned about the social implications of innate individual differences (Pinker, 2002). Another theory suggests that people are blind to nativism because innate mechanisms are incapsulated, and as such, they are opaque to introspection (Carruthers, in press; Cosmides & Tooby, 1994). While these results could offer a partial explanation for our findings, they fail to explain why these biases are selective to innate knowledge, specifically.

Another possibility is that participants’ reactions are informed by related life experience suggesting that cognitive traits, such as language and math, are learned. Although this possibility cannot be ruled out, we note that daily experiences also present evidence for the learning of motor skills (e.g., riding a bike, typing, and playing a musical instrument). Additionally, it is unclear why people would maintain this position in the face of explicit information suggesting that the relevant traits are innate, and why they would extend it to a nonhuman (i.e., less familiar) species. One could respond by saying that people discount evidence for innate knowledge because they question its reliability; perhaps they assume that cognitive traits are immaterial, so they cannot be measured precisely. Such beliefs, to be sure, are unjustified, as the information presented to participants (in Experiments 6–8) indicates that such traits are innate. Moreover, modern neuroscience tells us *all* psychological traits—cognitive or not—correspond to physical brain states. So, this explanation begs the question of *why* people believe that cognitive traits are immaterial, and how this presumption is linked to the bias against innate knowledge.

In a fourth explanation, the bias against innate knowledge is at the core of human cognition itself (Berent, in press). Past research suggests that people (including young infants) are intuitive *Dualists* (Bloom, 2004). We view objects as material entities, operating according to the laws of physics (Spelke, 1994; Spelke & Kinzler, 2007), but we attribute the actions of agents to immaterial mental states—their beliefs, knowledge, and goals (Onishi & Baillargeon, 2005; Woodward, 1998). It is precisely because mental states are presumed immaterial that children and adults believe they are more likely to transfer to the afterlife (Bering & Bjorklund, 2004) and migrate from one body to another (Cohen & Barrett, 2008; Cohen, Burdett, Knight, & Barrett, 2011) relative to noncognitive traits.

Other results indicate that people are also *Essentialists* (Gelman, 2003; Keil, 1986). A careful reading of the Essentialism literature further suggests that the essence of living things must be material. It is this presumption that explains why children attribute a doggy’s brown color to a tiny *piece of matter* transmitted from its mother (Springer & Keil, 1991), why they believe that the essence of a fossilized animal resides in a specific physical *location* (at its center, Newman & Keil, 2008), and why infants expect animate agents to have a material “*insides*” (Setoh, Wu, Baillargeon, & Gelman, 2013). Discreteness and localization are the defining features of chunks of matter.

These two principles of core cognition—Dualism and Essentialism—collide in reasoning about innate knowledge. If cognitive traits are immaterial (per Dualism) and innate traits are material (per Essentialism), then cognitive traits cannot be innate.

This proposal explains both why people readily assume the innateness of noncognitive traits and why they are resistant to innate knowledge. Noncognitive traits (sensations, motor traits, and emotions) can be easily mapped to material bodily states, so a mechanism for inheritance is easy for us to identify. But if, in our eyes, cognition (specifically, knowledge) is immaterial, then it is devoid of the potential for physical inheritance. A bias against innate knowledge, then, could well be the unintended casualty of the collision between two old evolutionary forces that shape human reasoning about the physical, psychological, and natural worlds—Dualism and Essentialism. If so, our resistance to innate knowledge could be in our nature. When it comes to knowledge, antinativism could be innate.

A series of experiments from our lab is in line with this possibility (Berent et al.,2019a). We found that (a) people consider cognitive traits (from Experiments 1–2) as less material than noncognitive traits (in line with Dualism) and (b) they consider innate traits (i.e., those that define human essence) more material compared to when the same traits are presented as acquired (in line with Essentialism). Moreover, reasoning about innateness can be altered by manipulating Dualism and Essentialism: people become more likely to view cognitive traits as innate when they are prompted to think about minds and bodies as one or the same (in line with Physicalism) compared to situations presenting minds and bodies as distinct (per Dualism). Additionally, when led to believe that the relevant traits are materially represented in the brain (per Essentialism), participants are more likely conclude that the traits are innate (compared to when the traits lack brain instantiation).

A second set of experiments (Berent, Feldman Barrett, & Platt, 2019) demonstrate the opposite biases for emotions—traits that are readily linked to the material body. We found that (a) participants assume that emotions are innate and embodied (facially and internally); (b) when told that emotions are localized in the brain (i.e., embodied), people conclude that emotions are innate; and (c) this naïve belief persists even when people are explicitly informed that the emotions in question are in fact acquired. Together, Dualism and Essentialism explain both our negative bias against innate knowledge as well as our positive bias to presume that emotions are innate.

Our present results cannot determine *why* people are resistant to innate knowledge. However, the findings do suggest that people are systematically biased in reasoning about the origins of knowledge. These conclusions shed new light on human nature and suggest caution in the scientific evaluation of innate knowledge.

## FUNDING INFORMATION

This research was supported in part by the Humanities Fellowship (to IB) from Northeastern University.

## AUTHOR CONTRIBUTIONS

IB: Conceptualization: Lead; Formal analysis: Lead; Methodology: Lead; Writing – Original Draft: Lead; Writing – Review & Editing: Lead. MP: Data curation: Supporting; Methodology: Supporting; Writing – Review & Editing: Supporting. GMS: Data curation: Supporting; Writing – Review & Editing: Supporting.
