# Supplementary material for: People’s Intuitions About Innateness
Source: Open Mind (Camb). 2019 Oct 1;3:101–14. doi: 10.1162/opmi_a_00029 (PMC8412331; doi:10.1162/opmi_a_00029)
Supplement: Supplementary file 1 [file opmi-03-101-s001.pdf]

https://www.mitpressjournals.org/doi/10.1162/opmi_a_00029

# *People's intuitions about innateness*

## *Supplementary Materials*

Iris Berent[1]
i.berent@neu.edu

Melanie Platt[1]
me.platt@northeastern.edu

Gwendolyn Sandoboe[1]
sandoboe.g@husky.neu.edu

[1]Department of Psychology
Northeastern University

**Address for correspondence**
Iris Berent
Department of Psychology
Northeastern University
125 Nightingale Hall
360 Huntington Ave.
Boston MA 02115
i.berent@neu.edu
Phone: (617) 373 4033
Fax: (617) 373-8714

https://www.mitpressjournals.org/doi/10.1162/opmi_a_00029

# Supplementary Methods

Participants. Experiments 1-8 each employed a unique group of participants. Participants were all adult native English speakers who were reportedly free of language and reading disorders and had not taken any advanced courses in psychology (beyond an introductory course). Participants had also reportedly not taken any advanced courses in linguistics (100%), and many had not taken advanced courses in biology (84%). Of all participants, 33% reported completing high school, 57% reported completing college, and 11% reported completing a graduate school program.

Experiments 1-8 were run over a period of two years. Having noticed that participants in later studies exhibited impossibly fast responses, in Experiments 3-8 (run later), participants were also required to answer a general comprehension question and to have spent a minimum of 200 s performing the experiment (including reading and responding to the consent form instructions and four vignettes); this requirement was added to ensure that participants were human agents who had attended to the task. All participants signed an informed consent, and the research protocol was approved by the IRB at Northeastern University.

Sample sizes were determined by extensive pilot work. Given those results, we expected a sample of N=20 to yield modest to large effect sizes in most within-subjects manipulations of trait used (in Experiment 1-5 and 7); the only exception was Experiment 6 (with bird traits), where the sample size of N=40 was determined by previous pilot experiments. Experiment 8 likewise used a sample size of N=40 as it included only a single trait pair, manipulated between subjects.

In all figures, error bars are calculated across participants. Confidence intervals for the difference between the means refer to the difference between cognitive and non-cognitive traits.

## Experiment 1

Materials. The materials corresponded to a randomized list of 80 traits. Cognitive traits (adapted from Pinker 2002) corresponded to behaviors that are broadly documented by ethnographers in many human communities, so they could be plausibly considered innate. Emotive traits were adapted from Pinker (2002) and from Lindquist et al.'s (2013) list of essentialist traits; motor traits were likewise selected to reflect universal human capacities. The descriptions of the three trait types were matched for length (M=4.2, SD=1.96; M=4.4, SD=1.14; M=4.2, SD=1.06; for cognitive, emotive, and motor traits, respectively). The traits were presented in a randomized order.

Procedure. One group of participants classified the traits into one of three categories: thinking, emotions, or actions. Participants were provided with a brief description of each category. They were also told that some behaviors include multiple components and advised to "classify the behavior according to the component that you judge to be most important".

A second group evaluated the propensity of the traits to emerge spontaneously in a desert island situation. To make the task more concrete, participants were invited to imagine a situation in which a group of infants were raised on a desert island, fully cared for, but devoid of the opportunity to watch these behaviors in their caregivers. Participants were asked to indicate how likely these people would be to exhibit each trait once they matured into adults and express their rating on a 1-7 scale (1= very unlikely; 7= very likely). The instructions to this and all subsequent experiments are provided in Appendix 2.

## Experiment 2

Materials**.** The materials consisted of a randomized list of 32 traits (16 cognitive, 16 non-cognitive), reflecting behaviors that have been documented in early infancy (e.g., Meltzoff & Moore, 1977; Spelke & Kinzler, 2007; Wurth, 1966; Háden, Németh, Török, & Winkler, 2015). The descriptions of the traits were matched for length (M=9.12, SD=3.44; M=8.33, SD=1.86; M=8.4, SD=3.01, for cognitive, sensory, and motor traits, respectively).

Procedure. As in Experiment 1, participants in the "trait classification" task were asked to classify traits into three categories and given a brief description of each category (e.g., action behaviors engage the body in motions—behaviors such as moving one's hands or legs). Participants were also informed that some traits might have multiple components and advised to classify the trait according to the component judged to be most important.

## Experiments 3-4

The trait list in Experiments 3-4 was as in Experiments 1-2, respectively. The procedure asked participants to indicate whether or not the traits are inborn. We used the term inborn (rather than innate) in order to mitigate against irrelevant political connotations of "nativism".

Participants were informed that "Inborn traits are ones that develop in humans/infants spontaneously. Some of these traits (e.g., having five fingers) are present in birth, but others (e.g., facial hair in men) can appear later in development. All inborn traits, however, emerge in the typical course of development, even if an individual has never had the opportunity to witness these behaviors in other people. Are each of the traits below inborn in humans/infants?".

## Experiment 5

Materials. The materials consisted of four vignettes, detailing four published experiments from the infant cognition literature. The language experiment gauged infants' preference for well-formed syllables like *blog* over ill-formed syllables like *lbog* (based on Gómez et al., 2014); the numeric cognition experiment examined whether infants detect the congruence between the number of auditory syllables and visual dots (based on Izard, Sann, Spelke, & Streri, 2009); the moral task examined whether infants prefer "helpers" over "hinderers" (based on Hamlin, Wynn, & Bloom, 2010); finally, the emotional task examined whether infants prefer happy faces to angry faces (based on Datyner, Henry, & Richmond, 2017).

https://www.mitpressjournals.org/doi/10.1162/opmi_a_00029

Some of the experimental descriptions were modified to simplify the tasks and to render them more comparable to each other. For example, the numerosity judgment task was modified to compare sets of two and four sounds/dots (rather than the larger sets in Izard et al., 2009) because we were concerned that laypeople might believe the infants are unable to compute large numerosities. Similarly, we modified the language experiment to use a behavioral looking time measure (rather than the near infrared spectroscopy, in Gómez et al., 2014) to render this task comparable to the other experiments (which likewise used looking time measures).

Each such description detailed the research question addressed by each experiment (e.g., can infants recognize the abstract number of objects and events?), the manner in which it was evaluated (e.g., the comparison of two sounds/two balls with two sounds/four balls), and the predicted link between infant behavior and the relevant trait (e.g., "if newborn infants can extract the abstract number of things/events, then they will prefer the number of sounds to match the number of visual objects. Accordingly, infants will look at the visual display longer if the number of objects matches the number of sounds (e.g., two syllables/two balls; four syllables/four balls) compared to when the number mismatches (e.g., two syllables/four balls or four syllables/two balls)").

## Experiment 6

Materials. The materials consisted of two pairs of matched vignettes, featuring cognitive and motor traits of various bird species. One pair featured behaviors related to singing: the structure of the swamp sparrow's song and the quail's head-bobbing behavior (for cognitive vs. motor traits, respectively). The other pair featured behaviors related to flying—the capacity of the bunting to reckon its migratory path and the zebra finch's flying style.

Cognitive traits relied on information structure (e.g., the abstract aXb rule governing the swamp sparrow's song, see Balaban, 1988), and their description provided explicit cues for thinking (e.g., the reference to the bunting's navigation as "reckoning"); the matched motor traits described a motor activity and highlighted their physical demands on the body. For example, the Japanese quail's head bobbing was described as "complex movement" that "requires motor precision and muscle control as it is always performed at a specific range of frequencies" (based on Balaban, 1997). Within a pair, the corresponding cognitive and motor vignettes were closely matched for word length and for their narrative structure. For example, the matched "singing" vignettes each began with matched introductory statements (*The swamp sparrow bird is a gifted composer* vs. *The Japanese quail is an agile dancer*) followed by a general description of the trait in question (e.g., *Male birds attract females by producing a characteristic song with a distinctive structure that is highly abstract* vs. *The male bird attracts the female by producing a characteristic head movement of a particular sequence of* movements). Subsequent sentences were likewise closely matched across the cognitive and motor pair members.

The four vignettes (2 cognitive/noncognitive x 2 pair) were arranged in four lists, counterbalanced for order (via a Latin square), and each list was assigned to 10 participants. Thus, trait type and vignette pair were each manipulated within participants.

https://www.mitpressjournals.org/doi/10.1162/opmi_a_00029

## Experiment 7

Materials. The materials consisted of matched vignettes, featuring cognitive and motor traits of various alien species. As in Experiment 6, the cognitive and motor vignettes were arranged in pairs, matched closely for word length and for their narrative structure as described above.

Cognitive traits relied on information structure (e.g., the abstract aXb rule governing the alien's light-signaling communication system), and their description provided explicit cues for thinking (e.g., the reference to the alien's navigation as "reckoning"); the matched motor traits described a motor activity and highlighted their physical demands on the body. For example, the alien's movement was described as a "complex locomotive style" in which the alien would "always move the four limbs in a circular forward motion, such that the left side of the body always precedes the right side".

The four vignettes (2 cognitive/noncognitive x 2 pair) were arranged in four lists, counterbalanced for order (via a Latin square), and each list was assigned to 5 participants. Thus, trait type and vignette pair were each manipulated within participants.

## Experiment 8

Procedure. Participants were invited to reason about a hypothetical desert island situation. Here, participants were introduced to Dana, a hearing child born to Deaf parents. Her loving parents are said to care for all her needs, but are unable to establish any linguistic communication with her, as they do not use a language (either spoken or signed). Participants were further told that Dana attends a daycare for children who are likewise deprived of a language, and caretakers who are Deaf (and do not use any language). Finally, participants were asked to reason what would happen if these children were to spontaneously generate new linguistic forms (either a word, or a sentence, for the motor and cognitive traits, respectively): would the child be more likely to form the preferred sequences like *blog/dogs bark* or the dispreferred ones, like *lbog/bark dogs*? Participants were instructed to indicate their response on a 1-7 scale (1=definitely choose *lbog/bark dogs*; 7=definitely choose *blog/dogs bark*).

Half of the participants were assigned to the cognitive vignette; the other half read the non-cognitive vignette. Thus, trait type is manipulated here between subjects.

## References


Balaban, E. (1988). Bird song syntax: learned intraspecific variation is meaningful. *Proceedings Of The National Academy Of Sciences Of The United States Of America, 85*(10), 3657-3660.

Balaban, E. (1997). Changes in multiple brain regions underlie species differences in a complex, congenital behavior. *Proc Natl Acad Sci U S A, 94*(5), 2001-2006.

Datyner, A., Henry, J. D., & Richmond, J. L. (2017). Rapid facial reactions in response to happy and angry expressions in 7-month-old infants. *Developmental Psychobiology, 59*(8), 1046-1050. doi:10.1002/dev.21575

https://www.mitpressjournals.org/doi/10.1162/opmi_a_00029

Gómez, D. M., Berent, I., Benavides-Varela, S., Bion, R. A. H., Cattarossi, L., Nespor, M., & Mehler, J. (2014). Language universals at birth. *Proceedings of the National Academy of Sciences, 111*(16), 5837-5341. doi:10.1073/pnas.1318261111

Háden, G. P., Németh, R., Török, M., & Winkler, I. (2015). Predictive processing of pitch trends in newborn infants. *Brain Research, 1626*, 14-20. doi:10.1016/j.brainres.2015.02.048

Hamlin, J. K., Wynn, K., & Bloom, P. (2010). Three-month-olds show a negativity bias in their social evaluations. *Developmental Science, 13*(6), 923-929. doi:10.1111/j.1467-7687.2010.00951.x

Izard, V., Sann, C., Spelke, E. S., & Streri, A. (2009). Newborn infants perceive abstract numbers. *PNAS, Proceedings of the National Academy of Sciences of the United States of America, 106*(25), 10382-10385.

Lindquist, K. A., Gendron, M., Oosterwijk, S., & Barrett, L. F. (2013). Do people essentialize emotions? Individual differences in emotion essentialism and emotional experience. *Emotion (Washington, D.C.), 13*(4), 629-644. doi:10.1037/a0032283

Meltzoff, A. N., & Moore, M. K. (1977). Imitation of facial and manual gestures by human neonates. *Science (New York, N.Y.), 198*(4312), 75-78.

Pinker, S. (2002). *The blank slate: The modern denial of human nature*. New York: Viking.

Spelke, E. S., & Kinzler, K. D. (2007). Core knowledge. *Developmental Science, 10*(1), 89-96.

Wurth, C. W. (1966). Apgar test for the neurological assessment of newborns. *Cereb Palsy J, 27*(1), 5-7.

https://www.mitpressjournals.org/doi/10.1162/opmi_a_00029

# Appendix 1
# Materials in Experiments 1-8
## Experiments 1 and 3: Adult Traits

| Number | Category | Trait |
|---|---|---|
| 1 | Emotion | Anger in response to hostility |
| 2 | Emotion | Love for one's family |
| 3 | Emotion | Contentment with one's life |
| 4 | Emotion | Disgust by feces |
| 5 | Emotion | Excitement towards an opportunity |
| 6 | Emotion | Fear of danger |
| 7 | Emotion | Happiness at the birth of one's child |
| 8 | Emotion | Joy of being |
| 9 | Emotion | Pride in one's accomplishments |
| 10 | Emotion | Sadness from a friend's death |
| 11 | Emotion | Shame from one's shortcomings |
| 12 | Emotion | Surprise at an unexpected event |
| 13 | Emotion | Trust in one's family |
| 14 | Emotion | Jealousy towards a lover |
| 15 | Emotion | Envy at a competitor's success |
| 16 | Emotion | Empathy towards a person in need |
| 17 | Emotion | Admiration for wisdom |
| 18 | Emotion | Pain from witnessing illness and death |
| 19 | Emotion | Hope for a better future |
| 20 | Emotion | Affection towards others |
| 21 | Motor | Gripping objects by hand |
| 22 | Motor | Sitting down to relax |
| 32 | Motor | Walking to move around |
| 24 | Motor | Running when in a hurry |
| 25 | Motor | Kicking with one's feet |
| 26 | Motor | Lifting objects with hands |
| 27 | Motor | Stretching one's muscles |
| 28 | Motor | Licking with one's tongue |
| 29 | Motor | Yawning when tired |
| 30 | Motor | Breathing heavily after exertion |
| 31 | Motor | Squatting down |
| 32 | Motor | Trembling at cold temperatures |
| 33 | Motor | Tickling a child to make them laugh |
| 34 | Motor | Touching other people with one's hands |
| 35 | Motor | Smelling the scent of food |
| 36 | Motor | Sleeping to restore one's energy |
| 37 | Motor | Seeing objects with one's eyes |

https://www.mitpressjournals.org/doi/10.1162/opmi_a_00029

| | | |
|---|---|---|
| 38 | Motor | Sniffling when one has a cold |
| 39 | Motor | Crying at sad events |
| 40 | Motor | Dancing to a rhythm |
| 41 | Cognitive | Recalling past events |
| 42 | Cognitive | Judging one’s options |
| 43 | Cognitive | Distinguishing between right and wrong |
| 44 | Cognitive | Reflecting on one’s past and future |
| 45 | Cognitive | Having self control of one’s own behavior |
| 46 | Cognitive | Speculating about the possible outcomes of events |
| 47 | Cognitive | Making jokes |
| 48 | Cognitive | Thinking about magic |
| 49 | Cognitive | Using metaphors |
| 50 | Cognitive | Mourning the dead |
| 51 | Cognitive | Observing rituals |
| 52 | Cognitive | Overcoming a fear |
| 53 | Cognitive | Recognizing taboos |
| 54 | Cognitive | Recognizing relations among kin |
| 55 | Cognitive | Interpreting others' behaviors |
| 56 | Cognitive | Symbolic reasoning |
| 57 | Cognitive | Making comparisons |
| 58 | Cognitive | Keeping track of time |
| 59 | Cognitive | Planning for the future |
| 60 | Cognitive | Recognizing melodies |
| 61 | Cognitive | Keeping track of people’s age |
| 62 | Cognitive | Forming sentences |
| 63 | Cognitive | Forming words |
| 64 | Cognitive | Abstract reasoning |
| 65 | Cognitive | Having preferences concerning aesthetics |
| 66 | Cognitive | Attributing human qualities to inanimate objects |
| 67 | Cognitive | Having a belief in the super-natural |
| 68 | Cognitive | Having beliefs about fortune and misfortune |
| 69 | Cognitive | Devising classification of body parts |
| 70 | Cognitive | Having classification of animals |
| 71 | Cognitive | Having classification of plants |
| 72 | Cognitive | Having classification of weather |
| 73 | Cognitive | Devising methods to heal the sick |
| 74 | Cognitive | Having a contrast between 'general' and 'particular' |
| 75 | Cognitive | Having a logical notion of 'and' |
| 76 | Cognitive | Having a logical notion of 'not' |
| 77 | Cognitive | Forming myths |
| 78 | Cognitive | Having a concept of 'person' |
| 79 | Cognitive | Having a preference for one's own children and close kin |
| 80 | Cognitive | Having norms about trade |

https://www.mitpressjournals.org/doi/10.1162/opmi_a_00029

# Experiments 2 and 4: Infant Traits

| Number | Category | Trait |
|---|---|---|
| 1 | Thinking | Expecting unsupported objects to fall down |
| 2 | Thinking | Expecting stationary objects to move only if contacted by other moving objects |
| 3 | Thinking | Expecting objects to move as connected wholes (e.g., without disintegrating) |
| 4 | Thinking | Understanding that objects still exist when occluded |
| 5 | Thinking | Expecting moving objects to continue their current trajectory (without changing direction) |
| 6 | Thinking | Recognizing the number of objects (e.g., two) |
| 7 | Thinking | Recognizing that, when one object is present, and another object is introduced, the total number of objects is two |
| 8 | Thinking | Recognizing that, when one of two objects is removed, only one object remains |
| 9 | Thinking | Recognizing that agents' actions are guided by goals |
| 10 | Thinking | Preferring agents who help others to those who hurt others |
| 11 | Thinking | Preferring human faces to nonhuman figures |
| 12 | Thinking | Preferring faces of one's own race to other races |
| 13 | Thinking | Preferring the gender of their primary caregiver |
| 14 | Thinking | Preferring human speech to non-speech sounds |
| 15 | Thinking | Preferring their mother's language to other languages |
| 16 | Thinking | Preferring foods from plants to foods from artifacts |
| 17 | Sensation | Contrasting low musical tones and high tones |
| 18 | Sensation | Contrasting the color red and the color blue |
| 19 | Sensation | Recognizing that the sound "ba" and the sound "pa" are different |
| 20 | Sensation | Distinguishing between the states of being awake and being asleep |
| 21 | Sensation | Preferring a good smell to a bad smell |
| 22 | Sensation | Distinguishing light color and dark colors |
| 23 | Action | Sticking out their tongue when they see an adult do the same |
| 24 | Action | Opening and closing their hands when they see an adult do the same |
| 25 | Action | Opening their mouth when they see an adult do the same |
| 26 | Action | Sucking on their thumb |
| 27 | Action | Making a fist with their hand |
| 28 | Action | Pulling away in response to pain |
| 29 | Action | Sneezing when they have a cold |
| 30 | Action | Coughing when they aspirate liquid |
| 31 | Action | Looking for something to suck on when their cheek is stroked |
| 32 | Action | Spreading out their arms and legs when they are startled |

https://www.mitpressjournals.org/doi/10.1162/opmi_a_00029

# Experiment 5: infant experiments

1. ***Number task***. This task examines whether newborn infants can recognize the abstract number of objects and events (e.g., 2, 3, 4). In the experiment, infants first hear *either* ***two*** or ***four*** spoken syllables (e.g., *tu-tu* vs. *tu-tu-tu-tu*)

   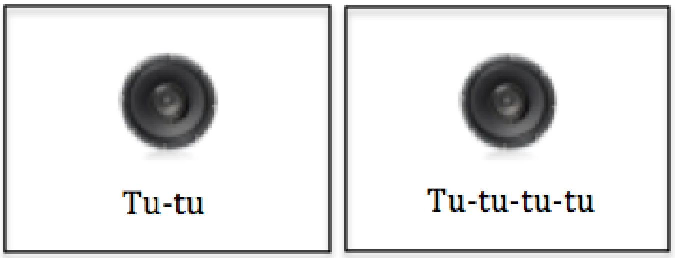
   

   Next, infants see *either* ***two*** or ***four*** circles.

   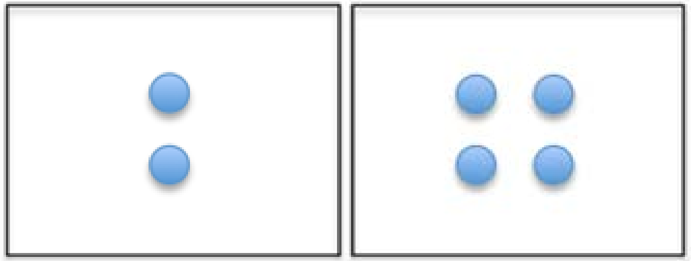

   The scientist reasons that if newborn infants can extract the abstract number of things/events, then they will prefer the number of sounds to match the number of visual objects. Accordingly, infants will look at the visual display longer if the number of objects matches the number of sounds (e.g., two syllables/two balls; four syllables/four balls) compared to when the number mismatches (e.g., two syllables/four balls or four syllables/two balls).

   In your opinion, would infants be likely to do so?

   a. Yes, infants will look longer at the matching vs. mismatching objects.
   b. No, infants will not distinguish between the matching and mismatching objects.

   Why?

2. ***Language task***. This task examines the linguistic capacities of newborn infants. Across the world's languages, syllables like *blog* are frequent, whereas syllables like *lbog* are rare. The researcher seeks to determine whether newborn infants prefer one syllable type to the other. To this end, she presents infants with a block of repeated syllables over a loudspeaker, either *blog, blog, blog*… or *lbog, lbog, lbog*….

   As the infants listen to these syllables, their eye gaze towards the loudspeaker is monitored. The researcher expects that if newborn infants prefer syllables like *blog*, then

they will look longer at the loudspeaker when presented with syllables like *blog* compared to *lbog*.

In your opinion, would infants be likely to do so?

a. Yes, infants will look longer at syllables like *blog* compared to *lbog*.
b. No, infants will not distinguish between syllables like *blog* compared to *lbog*.

Why?

3. ***Emotion task***. This task seeks to determine whether newborn infants recognize emotions. To this end, the researcher presents infants with two video displays. One display features a happy face; another features an angry face. The researcher expects that if newborn infants can recognize emotions, then they should look longer at the happy face compared to the angry face.

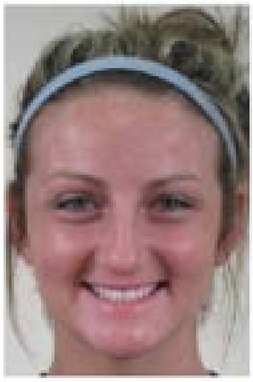

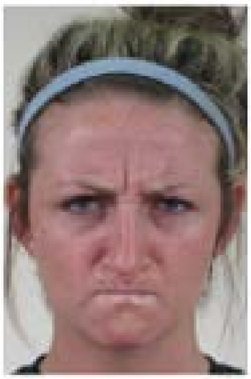

In your opinion, would infants be likely to do so?

a. Yes, infants will look longer at happy faces compared to angry faces.
b. No, infants will not distinguish between happy and sad faces.

Why?

4. ***Moral task.*** This task seeks to determine whether newborn infants have some rudimentary moral preferences. To this end, the researcher presents the infant with two video displays. Each display features two characters, consisting of geometric shapes. One character, a circle, seems to attempt at climbing up a hill. In one scenario, the second character (a triangle) helps the circle by pushing it up the hill; in another scenario, the second character (a square) hinders the circle climb by pushing it in the opposite direction (down the hill).

https://www.mitpressjournals.org/doi/10.1162/opmi_a_00029

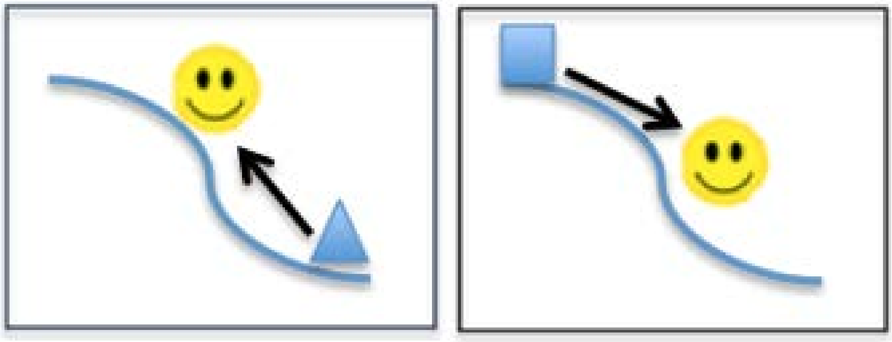

The next display presents infants with the triangle (helper) and square (hinderer). The researcher reasons that if infants have moral preferences, than they should prefer to look longer at the triangle (helper) than at the square (hinderer).

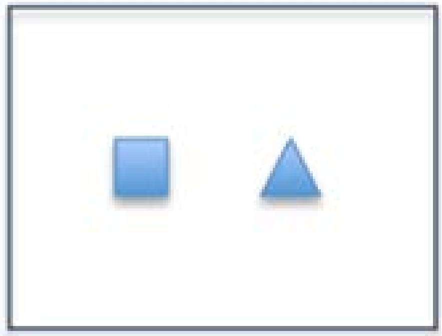

In your opinion, would infants be likely to do so?

a. Yes, infants will look longer at the triangle (helper) than at the square (hinderer)
b. No, infants will not look longer at the triangle (helper) than at the square (hinderer)

Why?

https://www.mitpressjournals.org/doi/10.1162/opmi_a_00029

# Experiment 6: Bird Traits

## Swamp sparrow song-cognitive

The swamp sparrow bird is a gifted composer. Male birds attract females by producing a characteristic song with a distinctive structure that is highly abstract. The song always begins with one particular sound (a) and ends in another (b), with various sounds permissible in between (X). Scientists describe the abstract structure of the song by the rule aXb. All male birds exhibit this exact abstract song structure from an early age, so scientists believe that the aXb rule is likely to be inborn in this species.

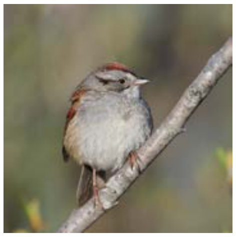

Suppose a group of fertilized sparrow eggs were separated from the parents and incubated in an isolated location until the chicks hatched. Those chicks would be fully cared for, but they would have no exposure to mature sparrows or their song. Once those chicks matured, how likely are they to produce the characteristic song structure? Please indicate your answer on a 1-7 scale (1=very unlikely; 7=very likely)

## Quail movement-motor

The Japanese quail is an agile dancer. The male bird attracts the female by producing a characteristic head movement of a particular sequence of movements. The male always bobs his head rapidly up and down. This complex movement requires motor precision and muscle control as it is always performed at a specific range of frequencies. All male birds produce the same complex head movement precisely in the same way, and they do so from an early age. Accordingly, scientists believe that this motor skill is likely to be inborn in this species.

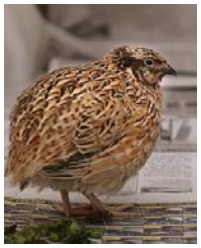

Suppose a group of fertilized quail eggs were separated from the parents and incubated in an isolated location until the chicks hatched. Those chicks would be fully cared for, but they would have no exposure to mature quails or their behavior. Once those chicks matured, how likely are they to produce the characteristic head bobbing? Please indicate your answer on a 1-7 scale (1=very unlikely; 7=very likely)

https://www.mitpressjournals.org/doi/10.1162/opmi_a_00029

## Indigo Bunting Navigation--cognitive

The tiny Indigo Bunting performs a remarkable intellectual feat. It can calculate its 2,000 mile navigational path from Central to North America. The bird is able to accomplish this complex reckoning by combining various pieces of information, including the position of the North Star, the strength of earth's magnetic field and the quality of sunlight at sunset. All members of the species navigate in this fashion, and they do so at an early age. Researchers believe that this intellectual capacity is likely to be inborn in this species.

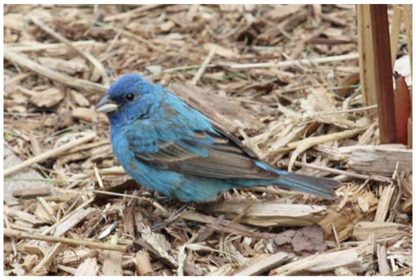

Suppose a group of fertilized Bunting eggs were separated from the parents and incubated in an isolated location until the chicks hatched. Those chicks would be fully cared for, but they would have no exposure to mature Buntings or their behavior. Once those chicks matured, how likely are they to successfully calculate their migration path? Please indicate your answer on a 1-7 scale (1=very unlikely; 7=very likely):

## Zebra Finch Bounding Flight --motor

The Zebra Finch bird is a gifted athlete that overcomes significant physical challenges. This small bird flies long distances thanks to a special "bounding" flight—a technique that alternates between short bursts of wing flapping with intervals in which the wings are folded against the body. This flying style reduces the demands on the bird's muscles, saves energy and allows it fly faster. All members of the species fly in this fashion, and they do so at an early age. Researchers believe that this physical capacity is likely to be inborn in this species.
.

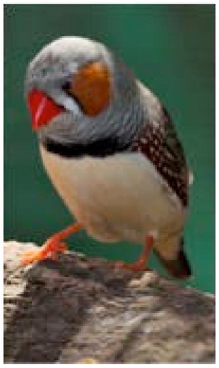

Suppose a group of fertilized Zebra Finch eggs were separated from the parents and incubated in an isolated location until the chicks hatched. Those chicks would be fully cared for, but they would have no exposure to mature Finches or their behavior. Once those chicks matured, how likely are they to successfully exhibit the "bounding" flight of the Zebra finch? Please indicate your answer on a 1-7 scale (1=very unlikely; 7=very likely):

https://www.mitpressjournals.org/doi/10.1162/opmi_a_00029

# Experiment 7: Alien Traits

**Alien Communication- (cognitive)**
Suppose an expedition to space has discovered an alien species living on Mars. Members of this species are highly intelligent. They communicate with each other using a complex light-signaling system, emitted from a special organ located on their head. This system allows individuals to exchange information, cooperate, and plan ahead. The system transmits information by altering the frequency of light pulses and their ordering. Members of this species always begin their messages with a rapid light pulse (a) followed by a longer pulse (b), with various types of permissible pulses in between (X). Scientists describe the abstract structure of the communication by the rule aXb. All members of this species use this system, and they begin doing so in their first year of life, so scientists believe that the aXb rule is inborn in this species.

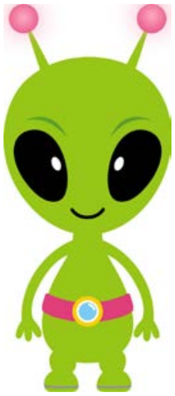

Suppose a group of alien infants were separated from their parents immediately after birth and transferred to an isolated location. Those infants would be fully cared for, but they would have no exposure to light signaling.

How likely is it that those infants would communicate using light signaling? Please indicate your answer on a 1-7 scale (1=very unlikely; 7=very likely).

**Alien Locomotion- (motor)**
Suppose an expedition to space has discovered an alien species living on Jupiter. Members of this species are quite agile. They move around using a complex locomotive style, generated by coordinating their four limbs. This form of locomotion allows individuals to efficiently navigate their terrain, which consists of vast distances and hills. Members of this species always move the four limbs in a circular forward motion, such that the left side of the body always precedes the right side. Scientists believe that this form of locomotion allows the species to save energy and reduce the demands on its muscles, move rapidly, and climb hills. All members of this species move around in this fashion, and they begin doing so in their first year of life, so scientists believe that the motor skill is inborn in this species.

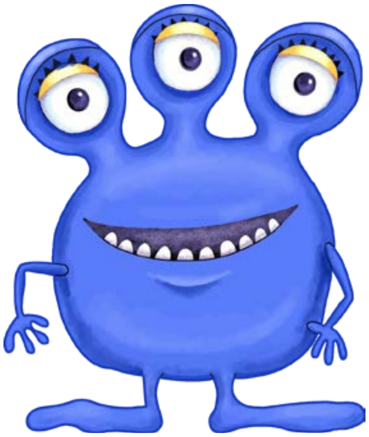

Suppose a group of alien infants were separated from their parents immediately after birth and transferred to an isolated location. Those infants would be fully cared for, but they would have no exposure to locomotion.

How likely is it that those infants would move in this particular way? Please indicate your answer on a 1-7 scale (1=very unlikely; 7=very likely):

https://www.mitpressjournals.org/doi/10.1162/opmi_a_00029

**Alien navigation- (cognitive)**

A team of scientists has discovered a species of aliens that performs a remarkable intellectual feat. Members of the species can calculate a 2,000 mile navigational path from one point on their terrain to another. The aliens are able to accomplish this complex reckoning by combining various pieces of information, including their planet's magnetic field and the position of other stars in their nightly sky. All members of the species can calculate their navigation in this fashion, and they begin doing so in their first year of life. Researchers believe that this intellectual capacity is inborn in this species.

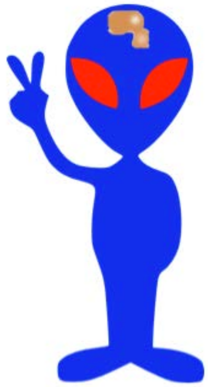

Suppose a group of alien infants were separated from their parents immediately after birth and transferred to an isolated location. Those infants would be fully cared for, but they would have no exposure to navigation.

How likely is it that those infants would be able to successfully calculate the navigational path? Please indicate your answer on a 1-7 scale (1=very unlikely; 7=very likely).

**Alien bounding flight- (motor)**

A team of scientists has discovered a species of aliens that overcomes some significant physical challenges. Members of the species can cover the long distances of their terrain thanks to a special "bounding" style of flying. This technique alternates between short bursts of flapping their limbs with intervals in which the limbs are folded against the body. This flying style reduces the demands on the alien's muscles, saves energy, and allows it advance faster. All members of the species fly in this fashion, and begin doing so in their first year of life. Researchers believe that this physical capacity is inborn.

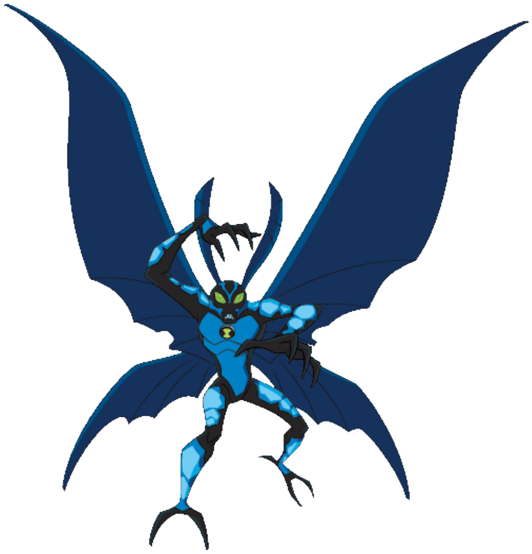

Suppose a group of alien infants were separated from their parents immediately after birth and transferred to an isolated location. Those infants would be fully cared for, but they would have no exposure to flying.

How likely is it that those infants would successfully fly using "bounding"? Please indicate your answer on a 1-7 scale (1=very unlikely; 7=very likely):

https://www.mitpressjournals.org/doi/10.1162/opmi_a_00029

# Experiment 8: Human Language

**Cognitive (syntax)**

When people come up with descriptions of actions, they are more likely to form sentences with certain word sequences as opposed to others. For example, people are more likely to make sentences like "dogs bark" than "bark dogs".

This is the case in most languages of the world, including English. Furthermore, studies have shown that infants prefer listening to the "dogs bark" over the "bark dogs" pattern. These results suggest that children develop this preference spontaneously, without learning.

Scientists think that patterns like "dogs bark" are more frequent because their abstract structure is simpler. When people form word patterns, their brain must align the words with an abstract sentence template. The optimal sentence template is "actor-action". Sentences like "dogs bark" fit this template perfectly, so their structure is easier for the brain to compute than the reverse order. For this reason, people naturally prefer forms like "dogs bark" relative to "bark dogs".
Consider now the following hypothetical thought experiment: Suppose that Dana is a child with normal hearing, born to Deaf parents. Her parents love her dearly and care for her, but they do not communicate with her using any language as, being Deaf, Dana's parents cannot speak, and they also do not use a sign language. Now, suppose that from the age of one month, Dana is sent to a day care where she spends most of her day with other hearing children in the same circumstances. Like Dana, these other children are raised by Deaf parents who do not use any language. Suppose further that the care-takers in this day care are likewise Deaf, and use no sign language, and for this reason, the children in the day care are not exposed to any language at all (spoken or signed). However, the children in the day care are free to communicate with each other in any manner they wish.

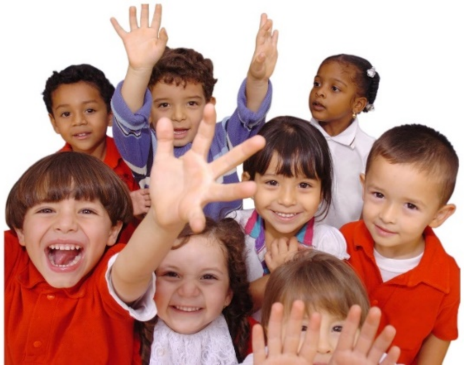

In your opinion, if the children were to spontaneously describe an action using language, would they be more likely to form sentences like "birds fly" or like "fly birds"? Please indicate your response on a 1-7 scale (1= definitely like "fly birds"; 2=probably like "fly birds"; 3=maybe like "fly birds"; 4=both equally likely; 5=maybe like "birds fly"; 6=probably like "birds fly"; 7=definitely like "birds fly")

**Motor (articulation)**

When people come up with names for new things, they are more likely to form words with certain sound sequences as opposed to others. For example, people are more likely to make up a word like "blog" than "lbog".

This is the case in most languages of the world, including English. Furthermore, studies have shown that infants prefer listening to the "blog" over the "lbog" pattern. These results suggest that children develop this preference spontaneously, without learning.
Scientists think that patterns like "blog" are more frequent because they impose fewer demands on the articulatory motor system. When people produce sound patterns, they must carefully coordinate movements of the lip and the tongue. Lip-tongue sequences (as in "bl") are coordinated naturally; the reverse order requires more articulatory effort (e.g., an additional articulatory sequence), so it is harder to articulate. For this reason, people naturally prefer forms like "blog" relative to forms like "lbog".
Consider now the following hypothetical thought experiment: Suppose that Dana is a child with normal hearing, born to Deaf parents. Her parents love her dearly and care for her, but they do not communicate with her using any language as, being Deaf, Dana's parents cannot speak, and they also do not use a sign language. Now, suppose that from the age of one month, Dana is sent to a day care where she spends most of her day with other hearing children in the same circumstances. Like Dana, these other children are raised by Deaf parents who do not use any language. Suppose further that the care-takers in this day care are likewise Deaf, and use no sign language, and for this reason, the children in the day care are not exposed to any language at all (spoken or signed). However, the children in the day care are free to communicate with each other in any manner they wish.

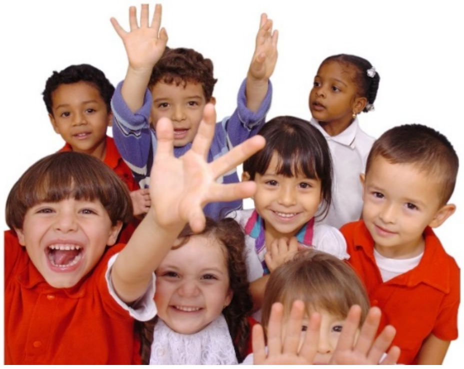

In your opinion, if the children were to spontaneously come up with a new name for an object, would they be more likely to create words like "blif" or like "lbif"? Please indicate your response on a 1-7 scale (1= definitely choose lbif; 2=probably choose lbif; 3=might choose lbif; 4=equally likely to choose either; 5=might choose blif; 6=probably choose blif; 7=definitely choose blif)

https://www.mitpressjournals.org/doi/10.1162/opmi_a_00029

# Appendix 2
# Instructions for Experiments 1-8

## Experiment 1: Adult Traits

**a. Classify Traits**

In this experiment, you will read short descriptions of human behaviors. Please read each description carefully. Then, classify each behavior into one of three types: thinking, action, or emotion.

Thinking behaviors extract and manipulate information—behaviors such as thinking, planning, and problem solving.

Action behaviors engage the body in motion—behaviors such as tapping your fingers or waving your hands.

Emotion behaviors involve one's feelings – behaviors such as feeling happy or sad.

Some behaviors may include multiple components. In such cases, classify the behavior according to the component that you judge to be most important. Please indicate your response by clicking on the appropriate button.

Thank you!

**b. Rate Innateness of Traits**

In this experiment, you will read a list of human traits. Please read each description carefully. Then, please determine how likely it is that a person would exhibit this trait spontaneously, even if they did not have the opportunity to learn it from others.

To be more concrete, consider the hypothetical situation of a group of infants raised on a desert island. These infants would be fully cared for, and their physical and emotional needs are all met, but they would not have the opportunity to observe any of these behaviors in their caregivers. As the infants mature into adults, how likely are these people to exhibit each trait? Please indicate your answer on a 1-7 scale (1= very unlikely; 7= very likely).

Thank you!

https://www.mitpressjournals.org/doi/10.1162/opmi_a_00029

# Experiment 2: Infant Traits

## a. Classify Traits

In this experiment, you will read short descriptions of various behaviors that an infant might exhibit. Please read each description carefully. Then, classify each behavior into one of these three types: thinking, sensation, or action.

Thinking behaviors extract and manipulate information—behaviors such as thinking, forming beliefs and setting goals.

Sensation behaviors involve the body's five senses— behaviors such as seeing, hearing etc.

Action behaviors engage the body in motions—behaviors such as moving one's hands or legs.

Some behaviors may include multiple components. In such cases, classify the behavior according to the component that you judge to be most important. Please indicate your response by clicking on the appropriate button.

Thank you!

## b. Rating innateness of traits

In this experiment, you will read short descriptions of various potential behaviors of infants. Please read each description carefully. Then, please determine how likely it is that an infant would exhibit this behavior spontaneously, without being shown or taught by a parent, caregiver, or any other person. Please indicate your answer on a 1-7 scale (1= very unlikely; 7= very likely).

Thank you!

# Experiment 3: Adult Traits (forced choice)

In this experiment, you will read a list of human traits. Please read each description carefully. Then, please determine whether or not these traits are **inborn** in humans.

Inborn traits are ones that develop in humans spontaneously. Some of these traits (e.g., having five fingers) are present in birth, but others (e.g., facial hair in men) can appear later in development. All inborn traits, however, emerge in the typical course of development, even if an individual has never had the opportunity to witness these behaviors in other people. Are each of the traits below inborn in humans?

Please indicate your answer as either 1=yes, this trait is inborn in humans OR 2=no, this trait is not inborn in humans.

https://www.mitpressjournals.org/doi/10.1162/opmi_a_00029

## Experiment 4: Infant Traits (forced choice)

In this experiment, you will read short descriptions of various potential behaviors of infants. Please read each description carefully. Then, please determine whether or not these traits are **inborn** in infants.

Inborn traits are ones that develop in humans spontaneously. Some of these traits (e.g., having five fingers) are present in birth, but others (e.g., facial hair in men) can appear later in development. All inborn traits, however, emerge in the typical course of development, even if an individual has never had the opportunity to witness these behaviors in other people. Are each of the traits below inborn in infants?

Please indicate your answer as either 1=yes, this trait is inborn in infants OR 2=no, this trait is not inborn in infants.

Thank you!

## Experiment 5: infant experiments

A researcher seeks to evaluate the mental capacities of newborn infants. To this end, the researcher presents a group of newborn infants with a variety of tasks. Here, we ask you to predict how the infants will perform on each task and provide a short explanation for your response.

## Experiment 6-7: Bird and Alien Traits

In this experiment, we will present you with several short vignettes. Each vignette describes a hypothetical scenario and invites you to think through this situation and express your opinion about it. Please read each story carefully and answer the questions as best you can.

Thank you!

## Experiment 8: Human language

In this experiment, we will present you with a short vignette that describes a hypothetical scenario and invites you to think through this situation and express your opinion about it. Please read each passage carefully and answer the questions as best you can.

Thank you!

# Appendix 3

## Experiment 3: Justifications provided by participants

| Task | Forced Choice Response | Description provided when asked "Why?" |
|---|---|---|
| Emotion | No | *They are to little to recognize the difference* |
| Emotion | No | *Again, I don't think their eyesight is developed enough. I think they'd look at any face* |
| Emotion | No | *Infants may look at a face longer because they do not recognize it or the expression.* |
| Emotion | Yes | *Pleasing* |
| Emotion | Yes | *facial rrecognition is all they have they dont know workds* |
| Emotion | Yes | *I think infants can distinguish between happy and sad faces.* |
| Emotion | Yes | *Newborns begin to orient to faces early* |
| Emotion | Yes | *Because they are stimulated by happiness* |
| Emotion | Yes | *Because infants could feel the happiness and friendly fae which they like to look more than the angry face that makes them want to run away.* |
| Emotion | Yes | *Newborns probably can understand emotion and the significance of facial expressions.* |
| Emotion | Yes | *THEY MAKE ALL KIND FACAIL IMPRESSIONS* |
| Emotion | Yes | *smiles attract people* |
| Emotion | Yes | *that is human nature* |
| Emotion | Yes | *They respond to emotion and touch at a young age. Their brains are programmed to feel many emotions.* |
| Emotion | Yes | *It is innate in humans to do so.* |
| Emotion | Yes | *It's an innate sense of positivity.* |
| Emotion | Yes | *The happy face is just more appealing especially for an infant.* |
| Emotion | Yes | *They will try to imitate the facial features* |
| Emotion | Yes | *because being happy is easier* |
| Emotion | Yes | *[no response]* |
| Number | No | *I think these situations are too complex for newborns. they are still in the developmental stage of judging pictures.* |
| Number | No | *They aren't aware of counting principles yet* |
| Number | No | *Infants will not be able to connect these two events as connected.* |
| Number | No | *They arenâ€™t developmentally understanding of things that are equal.* |
| Number | No | *Recognizing patterns across different objects would be too advanced for an infant.* |
| Number | No | *DONT THINK INFANT CAN UNDERSTAND THESE THINGS* |
| Number | No | *they are too young to distinguish them* |
| Number | No | *newborns don't see well* |
| Number | No | *I don't think newborns' sight is developed enough at that point to see the number of objects* |
| Number | No | *They are to little to recognize the difference* |
| Number | No | |

| | | |
|---|---|---|
| Number | No | *I don't think they can.* |
| Number | No | *I feel infants do not have that capability yet.* |
| Number | No | *It does not seem like a newborn would have this ability yet.* |
| Number | No | *they would not know* |
| Number | Yes | *color* |
| Number | Yes | *So, infants are more likely to focus on the number of objects in a picture as opposed to hearing a differentiation in language.* |
| Number | Yes | *I think infants will look longer at the matching vs. mismatching objects as they probably know the the matching like when we play with infants for the matching games.* |
| Number | Yes | *A newborns mind is extremely open to learning at this early stage.* |
| | | *because they will most likely have the number of sound and number of objects to match and that will keep there attention* |
| Moral | No | *they are to little to recognize the difference* |
| Moral | No | *Newborn infants are not able to make moral judgments* |
| Moral | No | *I donâ€™t think they will make that association.* |
| Moral | No | *I don't think an infant would make that connection.* |
| Moral | No | *DONT THIK THEY CAN TELL DIFFRENCE* |
| Moral | No | *They couldn't differentiate between the two shapes* |
| Moral | No | *I don't think we're born with a sense of morals. That is learned* |
| Moral | No | *I do not think the newborns have any concept of moral values at birth.* |
| Moral | No | *Infants can not see shapes because their brains are still developing.* |
| Moral | No | *too complex for newborn* |
| Moral | No | *complex* |
| Moral | No | *[no response]* |
| Moral | No | *I don't think they can.* |
| Moral | No | *I think they'd look longer at the smiley face.* |
| Moral | No | *square image has arrow pointing down which attracts more attention* |
| Moral | No | *Complex* |
| Moral | Yes | *Because the triangle is more complex* |
| Moral | Yes | *Since they feel the triangle is the helper.* |
| Moral | Yes | *I think infant would look longer at the triangle (helper) than at the square (hinderer) because the shape of triangle is pointy.* |
| Moral | Yes | *the shape* |
| Language | No | *they are to little to recognize the difference* |
| Language | No | *not interested* |
| Language | No | *This scenerio is too complex for infants.* |
| Language | No | *They will probably just look at it because they hear talking.* |
| Language | No | *I feel infants do not have that capacity.* |
| Language | No | *They may be attracted to any sound at all.* |
| Language | No | *I do not think the newborns are capable of differentiating between these kinds of sounds.* |
| Language | No | *I think they would look to the speaker the same amount of time.* |
| Language | No | *INFANT CANT DO THESE THINGS* |
| Language | No | *the word is more interested* |

| | | |
|---|---|---|
| Language | No | *[no response]* |
| Language | Yes | *natural sound* |
| Language | Yes | *I think the newborns are born with a sense of language understanding* |
| Language | Yes | *[no response]* |
| Language | Yes | *Infants are oriented to more familiar sounds* |
| Language | Yes | *I think if it sound like something they hear at home ( a syllable more common in their language like blog) than they will look to see who is speaking.* |
| Language | Yes | *The fewer syllables the more interested the infants are.It's a thing of simplicity.* |
| Language | Yes | *I think infants love to look at syllables like blog compared to lbog.* |
| Language | Yes | *Ibog sounds like two words* |
| Language | Yes | *easier to listen* |
